# Medical Items and News of Interest to the Physician

**Published:** 1869-10

**Authors:** 


### Of Interest to the Physician.

CULLED FROM THE STANDARD MEDICAL JOURNALS OF THE WORLD.

*Note:*

These formulæ are intended only for the regular practitioner of medicine, and should be employed only by him, or under his immediate supervision.

**Taste of Cod-Liver Oil Disguised.**

Spread the oil on toast, grate over it a little smoked salmon, herring, or ham. It can then be eaten with no more effort than salt mackerel.

**Chlorosis.**

Ten drops of Fowler's Solution and one thirtieth grain of strychnia, ter die, are given by Prof. W. A. Hammond in chlorosis.—*Record.*

**Dr. Brown-Sequard.**

The Parisian government intends building a *laboratorie* for this distinguished pathologist—wonder whether he will then advance on his price of $1,500 for informing a gentleman that he has a boil in his ear ?"

**Hydrocephalus.**

There is a child in Dayton, Ohio, aged 17 months, whose head measures 27 inches in the occipito-frontal circumference, and 19 inches from one meatus of the ear to the other, over the top of the head.

**Phenic Acid in Syphilis.**

The application of phenic acid, five parts to 100 of water, to syphilitic ulcers, is recommended by an Italian physician. The deuto-phosphate of mercury, one grain a day, continued for two months, in tertiary syphilis, is advocated by Dr. Fidele de Fiori.

**Ovariotomy at Twelve Years.**

At the meeting of the Société Impériale de Chirurgie, May 26, M. Jonson related an extraordinary case of ovarian tumor in a child 12 years of age. Ovariotomy was performed, and the patient recovered. The tumor weighed twenty-six pounds.

**Intermittent Fever.**

Dr. Jessier (Boston *Medical and Surgical Journal*), claims to have cured intermittent fever by carbolic acid, in a patient with whom quinine had failed. Three-fourths of a grain of the pure acid dissolved in twenty drops of water, were injected beneath the skin.

**A Prolific Child-Bearer.**

A physician of Tennessee reports, in the *Richmond and Louisville Medical Journal*, the case of a woman, only thirty-four years old, who has given birth to *twenty-four children.* She was not an American woman, by any means, (they don't do business in that way) but a negress.

**N. Y. State Inebriate Asylum.**

Since the 1st of May, 1867, 228 patients have been discharged from this Asylum, situated at Binghamton, N. Y. It is stated that 113 permanently reformed, after a single probationary trial; 11 have fallen after a first trial, and 4 after a second; but those are said to have overcome the habit of intemperance by returning to this Institution.

**Preserving the Cadaver.**

M. Devergie, at a late session of the Paris Academy of Medicine, read the results obtained in the School of Practical Anatomy by injecting bodies with a mixture of one part of Carbolic acid and three parts of glycerine. Bodies thus injected were preserved several months without emitting any offensive odor.—*Med. Record.*

**Sutures.**

We have tried all manner of sutures, metalic, gut, hair and prepared. Our experience is that common, white, saddler's silk boiled in bees-wax, and then rubbed perfectly smooth and regular, are by far the best sutures in use. We use thread so prepared in ovariotomy, staphylorraphy, and vesico-viginal-fistula, and find it superior to every other sort of suture.

**Amputation of Diseased Lung.**

The *Med. Press and Circular* says that Dr. Downing proposes to amputate a diseased lung by thrusting a hollow needle, with a valve at the outer extremity, between two of the ribs of the side affected, so as to reach the bag of the pleura. The valve is then opened and a small portion of warm air admitted into the cavity at each act of inspiration. By means of this ingress of air there will be a corresponding collapse in the lung, when expectoration or ejection of the pus occurs until the lung is completely emptied of its contents and firmly contracted against the dorsal vertebræ.

**Fee for Cataract.**

The *Med. Record* says that "Dr. Magni, Prof. of Ophthalmology in the University of Bologna, Italy, is to receive 100,000 francs, about $20,000 in gold, for an operation for cataract on a merchant residing in Lima, Peru. The expenses of the journey for himself and assistant are also paid by the Liman Merchant." Whew—would'nt we like to operate upon the remaining eye at same price!

**Cure of Carotid Aneurism.**

M. Rouge reports the following case to the Society of Surgery, Paris:

Patient 68 years old. Compression affected laterly, the thumb being placed against the anterior edge of the sterno-mastoid, the next three fingers under the posterior edge, and the artery being thus seized and compressed between them. Digital compression was continued seventeen days, for an average of seven or eight hours each day.—*Bost. Med. Jour.*

**Expulsion of Tape Worm.**

A physician writes us that he gave a woman æt 28, three compound cathartic pills at bed time. In the morning at 9 o'clock, having taken no breakfast, she swallowed an ounce of oil of turpentine in strong coffee. This was followed, in the course of two hours, by an ounce of castor oil. The worm, which measured 34 feet in length, made its appearance in the course of the evening.

**Treatment of Acute Rheumatism.**

Dr. Chadwick, of the Leeds General Infirmary, after much experience and a vast variety of changes, now relies upon bi-carbonate of potash, in twenty grain doses every four hours, and keeping the patient in blankets. The change from the sour perspiration and the alkaline condition of the urine may point to a less frequent administration of the remedy, but does not warrant him in changing it altogether. Since he adopted the sleeping in blankets, he believes he has had less endocardial and pericardial complication.—*Medical News.*

**Tertiary Syphilis.**

Prof. Gross' formula for tertiary syphilis is as follows:

℞.
Hyd. chlo. corros., gr. ij.
Potassii iodidi, ʒ ij.
Syrp. sarsa. Comp. f. ℥ iij.
M.

S.—Dessert spoonful ter die. shortly after meals.

**Delirium Tremens.**

The following formula has been found effectual in the hands of a friend who sends it to to the BISTOURY:

℞.
Chloroform, ℥ j.
Glycerine, ℥ ss.
Morphia, gr. ij.
M.

S.—One teaspoonful every half hour until sleep is produced.

**Chronic Duodenitis.**

Dr. John Mulvany (*Bost. Med. Jour.*) gives the following prescription:

℞.

Olei petrol., ℥ ss.

Chloroform, ʒ ss.

Tinct. Cardam. Co., ʒ j.

M.

From fifteen to twenty drops are dropped on sugar, and administered three hours after every meal.

**Constipation in Old Age.**

Dr. J. M. DaCosta (*Med. and Surg. Reporter*) recommends the following formula:

℞.

Podophyllin, ——

Ext. Belladonnæ, āā gr. 1-20.

Capsici, — — gr. ¼.

Pulv. Rhei, — — gr. j.

For one pill. To be taken ter die.

**A Quaint Prescription.**

Dr. Upham (*Boston Med. and Surg. Jour.*), has in his possession the subjoined old-fashioned prescription, sent by a famous London physician to John Winthrop, Governor of the Colony of Massachusetts Bay, A. D. 1643. It is called a "remedy against fevers, poysons, small-pox, the plague, and such like":

℞.

"In the month of March take Toades, as many as you will, alive; putt them into an earthan pott, so ye bottom may be uppermost; put charcoales round about it, and in ye open arye, *not in an house.* Sett it on fire; when cold take out ye toades and in iron mortar pound them well and *tearce* them (whatever that may be)—a black powder will result. Of this you may give a dragme inwardly in any affection. For prevention ½ a dragme will suffice. Moderate the dose according to ye strength and constitution of ye partie."

**Dysmenorrhœa.**

T. Hawkes Tanner, M. D., London, recommends the following:

℞.

Pot. bromidi, ʒ j.

Tinct. cantharidis, f. ʒ jss.

" cinnamoni, f. ʒ vj.

Aquae q. s. ad f. ℥ jss.

Dessert spoonful three times a day.

---

**Treatment of Chancres.**

Freeman J. Bumstead makes use of the following:

℞.

Hyd. chlor. mitis. gr. xxxvj.

Tinct. Opii, f. ʒ j.

Cerati simplicis, ℥ j.

M.

Apply to chancre by spreading salve upon a cloth.—*Med. and Surg. Reporter.*

---

**Epilepsy.**

℞.

Pot. iodidi, ʒ j.

Pot. bromidi, ℥ j.

Ammonii bromidi, ʒ ijss.

Pot. bicarbonatis, ℈ ij.

Infusi Calumbæ, f. ℥ vj.

M.

A teaspoonful before each meal, and three teaspoonfuls at bed time, with a little water, is prescribed by Brown-Sequard.—*Med. and Surg. Reporter.*

---

**Bronchitis in Children.**

Dr. J. M. DaCosta is in the habit of prescribing the following mixture, to be taken at one dose, every third hour:

℞.

Spts. ætheris nitrosi, m. x.

Ammoniæ carbonatis, gr. j.

Syrupi senegæ, m. xx.

" tolu, m. x.

Aquæ, m. xx.

M.

---

**Chronic Diarrhœa.**

℞.

Acidi gallici, gr. xij.
Tinct. cinnamomi, f. ʒ jss.
F. Tinct. Opii, m. viij.
Aquæ carui, q. s. ad. f. ℥ ij.
M.

Dose—Two teaspoonfuls for a child two years old, with chronic diarrhœa and irritable stomach.—*Ther. Bull. Med. and Surg. Reporter.*

**For Neuralgia.**

Procure the following ointment and rub the parts affected when the paroxysms of pain are coming on:

℞.

Veratriæ, gr. v.
Morphiæ Sul. gr. iij.
Adipis, ℥ j.
M.

---

**Gonorrhœa.**

℞.

Tinct. cubebæ, f. ʒ j.

Copaibæ, f. ʒ ss.

Liq. Potassæ, gtt. v.

Liq. morphæ sul. f. ʒ ss.

Aquæ cinnamomi,

Misturæ camphoræ,

Pulv. acaciæ, āā f. ʒ j.

Sacchari albi, gr. vij.

For one dose—Three times a day.

D. HAYS AGNEW,
*Med. and Surg. Reporter.*

---

**Substitute for Dover's Powder.**

The following powder is offered as a substitute for the old Dover powder, by an old practitioner writing to the Phila. *Med. and Surg. Reporter:*

℞.

Opii, pulv., ʒ j.

Ipecac, pulv., ʒ ss.

Camphor, pulv., ʒ ij.

Saccharum, ʒ jv.

Mix thoroughly.

It is recommended to be given in same dose as the Dover powder, to which it is regarded superior, in consequence of its non-nauseating qualities and pleasant flavor.

---

**Incipient Goitre.**

Dr. I. Waring-Curran says the following formula is used extensively in England:

℞.

Ammonii iodidi, gr. xi.
Spiritus Chloroformi, ʒ ij.
Aquæ camphoræ, ad, ℥ viij.

Cap. ℥ j., ter in die.

At the same time the following cerate is rubbed into the growth night and morning:

℞.

Ammonii iodidi,

Glycerinæ, āā ʒ ij.

Adipis benzoat, ℥ jss.

**Alkaline Treatment of Rheumatism.**

The *Practitioner* for March contains an able argument by Dr. H. W. Fuller, in favor of the alkaline treatment of rheumatism. He administers an ounce and a half of the alkaline carbonates, either with or without vegetable acids, during the first 24 hours. If the bowels are torpid, he gives at the same time, ten grains of colocynth and calomel pill at bed-time. The urine usually becomes alkaline in 24 hours, and the quantity of alkali is then reduced one-half daily until the fourth day, after which barely enough is given to keep the urine neutral. Cinchona or quinia are given after the fourth day, and aperients if necessary. The diet is restricted to beef-tea, milk, and soda water, and all solid food is absolutely forbidden, as also wine and spirits.

---

**Spina Bifida,**

M. Roux in France, has recently published in the *Bulletin de Therapeutique*, the case of a girl, six weeks old, presenting this deformity. The tumor hung from the extremity of the sacrum to the lower third of both thighs. The author first made an exploratory puncture, and removed about an ounce of limpid fluid. An assistant was desired to hold the tumor in such a manner as to occlude the opening into the spinal canal; the operator then injected an ounce of the following solution:

℞.

Distilled water, ʒ xj.

Tincture Iodine, ℥ iij.

Iod. Pot. ʒ iij.

M.

The liquid was left five minutes in the sac, the latter being kneaded with the hand of the operator. The solution was then withdrawn to the last drop of the exhausting agency of the syringe. This proceeding succeeded so well that, in a fortnight, there was a hard nucleus left no larger than a walnut. M. Roux attributes his success to the occlusion of the canal, and to the withdrawal of the very last drop of the injected fluid.

---

**Treatment of Scarlet Fever.**

Dr. Charles T. Thompson reports in the *London Lancet* his manner of treatment in scarlet fever as follows:—The patient is immersed in a warm bath in the early stage of the disease, and this is repeated frequently, or as often as the strength of the patient will allow. The first effect is to produce a soothing and refreshing feeling in the patient, to be followed soon by such an eruption upon the surface, of so vivid a color, and in such amount, as would astonish those who have never witnessed it. Thus one of the greatest dangers of this fearful disease—the suppression of the eruption—is escaped. The appetite generally returns after the first or second bath, and the strength of the patient is kept up by nutritious food. The bath prevents dissemination of the disease, by removing the excreta from the skin as soon as it is deposited. This treatment promotes cuticular desguamation. The body should be gently dried by soft linen cloths after the bath.

By this procedure the various secretions are deprived of their noxious properties, and the irritation of internal organs is quickly relieved, thus dissipating infection. Another benefit is that a very serious case is soon reduced to a mild one, and the patient recovers in less than half the usual time. Since Dr. T. has pursued this practice—during fifteen years—he has never lost a patient from scarlet fever.

---

**Dysentery.**

A physician writing to the Philadelphia *Med. and Surg. Reporter*, greatly extols the following formula in severe cases of dysentery:

℞.
Peach leaf syrup, ℥ jv.
Syrp. Epecac, ℥ ij.
Arom. syrp. rhubarb, ℥ j.
Acitate morphia, gr. viij.
M. Fiat Sol.

Sig:—Give one teaspoonful every two hours through the day, letting the patient rest undisturbed through the night.

He explains that the "peach leaf syrup" is not officinal, but can be easily made in the following manner: "Fill a kettle, of any size, with fresh, well matured peach leaves, and enough water to cover the leaves, weighting the leaves down, boil gently one hour, remove the leaves, strain the liquor, and while hot add sugar to make a very sweet syrup; and when nearly cool, add one ounce of peach brandy and two grs. gum-camphor to each quart of syrup. Let it remain in bulk 24 hours, then draw off and bottle for use."

We believe the formula to be a good one, as we have heard good reports of its use from physicians who use the peach leaf syrup.

### TRIBUTES TO VANITY.

WHAT THE JOURNALS SAY OF US.

The BISTOURY for July is out, filled to overflowing with a choice and interesting variety of reading matter.—*Northern Pennsylvanian*.

THE BISTOURY.—A Quarterly Medical Journal, published at Elmira, N. Y., is among the sprightliest of our exchanges. We never fail to read it, and advise our patrons to send fifty cents to the Editor, Dr. Up De Graff, and do likewise.—*Journal of Commerce*, Kansas City.

QUACKERY.—Dr. Up De Graff, at Elmira, N. Y., publishes a splendid little quarterly, which he calls the BISTOURY. It is truly a double-edged instrument, and dissects all forms of quackery and humbuggery without mercy. We advise everybody to subscribe for it—it is useful, instructive and cheap—only fifty cents a year.—*Echo*, Portland, Me.

The BISTOURY, for July, takes on the full proportions of a Health Journal. The Communications, Correspondence, Editorials, and clippings, are all even better than the last No. If you want a large dose of common sense for 50 cents, subscribe for the BISTOURY, Dr. T. S. Up De Graff, proprietor, Elmira, N. Y.—*Agitator*, Willsboro, Pa.

THE BISTOURY.—This is the title of a neatly printed quarterly journal, published at Elmira, by Thad. S. Up De Graff, M. D. We have perused Dr. Up De Graff's magazine, hastily, we confess, but with interest, and believe it to be a practical and useful work. Price only 50 cents per annum, in advance.—*Standard*, Williamsport, Pa.

A SENSIBLE JOURNAL.—The BISTOURY, edited by Thad. S. Up De Graff, M. D., at Elmira, N. Y. It has a circulation in every State in the Union, as it deserves to have—a large number are taken in this city, and are greatly enjoyed by reason of their exposition of all manner of charlatanism, We wish its Editor abundant success.—*Advocate*, New Orleans, La.

The BISTOURY, a quarterly journal, of twenty-eight pages, devoted to the exposition of Charlatanism in Medicine and the education of the people upon medical subjects, has found its way to our table, and from a hasty perusal of its pages, we are of the opinion that it should find its way into every family in the land. Its low price—50 cents per year—should enable it to do so. It is edited by Thad. S. Up De Graff, M. D., and published at Elmira, N. Y.—*Democrat*, Honesdale, Pa.

The BISTOURY, by Thad S. Up De Graff, M. D. comes to us much improved. It is now published in pamphlet form, and makes a very neat appearance. The object of the BISTOURY is to enlighten the family circle on medical subjects. Published quarterly, 50 cents a year. The Editor, with whom we have the pleasure of an acquaintance, has made a few visits South, and has performed several successful ovariotomy operations here.—*Friend*, Sparta, Ga.

THE BISTOURY.—Thad. S. Up De Graff, Editor. A quarter of months ago, the BISTOURY was changed to pamphlet or magazine form, and it was then announced that it would appear quarterly. The subscription price was placed at fifty cents per annum, when before, the circulation was gratuitous. The BISTOURY now appears a second time under the new arrangement; and, to all appearances, the enterprise of its Editor is a success. His editorial is full of congratulation to that effect, rather jubilantly exclaiming: "From a circulation of three thousand to that of more than *twenty thousand!* If so much can be accomplished in three years, how much will we grow in the *next* three? We havn't time to solve the problem now, but will assure the many patrons of the BISTOURY that we thank them heartily for responding so handsomely to our subscription price. We can now write to some purpose, and will continue to improve and enlarge our journal as fast as the subscriptions of our friends will allow us to. Give us the means to defray the expenses of publishing this journal, and we will give you the best and cheapest family magazine in America." The table of contents of the July number comprises a communication from B. H. Pratt, A. M., who is one of the most promising writers ever connected with an Elmira journal of any character. The subject of his article in the July number is, "What to Drink, and How, and Where, and When." The journal also contains other able communications, which, with correspondence, clippings and editorials, make up a number of unsurpassed interest. We do not hesitate to characterize the BISTOURY as a spicy and valuable journal—having no superior in its line.—*Elmira Daily Gazette*.

